# l-Ascorbyl-2-phosphate attenuates NF-κB signaling in SZ95 sebocytes without affecting IL-6 and IL-8 secretion

**DOI:** 10.1007/s00403-015-1565-z

**Published:** 2015-04-18

**Authors:** Hiroshi Ikeno, Mara Apel, Christos Zouboulis, Thomas A. Luger, Markus Böhm

**Affiliations:** Ikeno Clinic of Dermatology and Dermatologic Surgery, Ginza 1-14-4, 3F, Chuo-ku, Tokyo, Japan; Department of Dermatology, University of Münster, Münster, Germany; Department of Dermatology, Venereology, Allergology and Immunology, Dessau Medical Center, Dessau, Germany

**Keywords:** Sebocytes, Ascorbyl-2-phosphate, Cytokines, Acne

## Abstract

Acne is the most common inflammatory skin disease. Interleukin-1 (IL-1) is at the beginning of the cytokine signaling cascade and may be involved in the pathogenesis of this disorder. It activates redox-sensitive transcription factors, which induce IL-6 and IL-8 expression. Interestingly, l-ascorbyl-2-phosphate (APS) was shown to have beneficial effects in patients with acne vulgaris. The mechanism of action of this agent remains unknown. Here, we investigated if APS attenuates IL-1β- or TNF-α-mediated IL-6 and IL-8 expression in SZ95 sebocytes, whereas TNF-α was used as control. We also explored NF-κB activation which is known to orchestrate IL-1β- and TNF-α-mediated cytokine expression in many cell types. Both IL-1β and TNF-α increased IL-6 and IL-8 mRNA expression in SZ95 sebocytes. However, only IL-1β induced IL-6 and IL-8 secretion. IL-1β but not TNF-α activated NF-κB canonical signaling as demonstrated by Iκ-Bα phosphorylation and degradation as well as by nuclear accumulation of NF-κB/p65. Concomitant treatment of SZ95 sebocytes with APS attenuated the effect of IL-1β and TNF-α on IL-6 and IL-8 gene expression as well as on IL-1β-mediated NF-κB signaling. In contrast, APS failed to reduce IL-1β-mediated IL-6 and IL-8 secretion, presumably by maintained IL-1β-mediated p38 activation, which is known to control IL-8 secretion. Our findings shed light into the impact of IL-1β on the inflammatory cytokine response and its molecular mechanisms in human sebocytes. Our data further suggest that the beneficial effect of APS in acne patients involves attenuation of NF-κB signaling but not reduction of IL-6 or IL-8 secretion.

**Electronic supplementary material:**

The online version of this article (doi:10.1007/s00403-015-1565-z) contains supplementary material, which is available to authorized users.

## Introduction

Acne is considered the most common inflammatory skin disorder. It is estimated that about 93 % of adolescents suffer from acne vulgaris [9], the most common subtype of acne. Albeit our current therapeutic armamentarium against acne vulgaris encompasses a panel of usually very effective topical and/or systemic treatments other subtypes of acne such as acne conglobata, smokers’ acne or acne tarda can still be a therapeutic challenge.

Elucidating the key events that lead to an uncontrolled inflammatory response of the pilosebaceous unit will be crucial to optimize our current therapeutic options and to tailor new treatments against acne. Among the various proinflammatory mediators interleukin-1 (IL-1) is the most upstream player within the cytokine cascade. On the other hand, tumor necrosis factor-α (TNF-α) seems to play a minor role in acne inflammation. Nevertheless both cytokines are robust inducers of IL-6 and IL-8 gene expression [5, 25] and propagate an initial inflammatory response of the pilosebaceous unit in acne [21]. IL-1α-like bioactivity is present in the majority of open comedones [16]. Using a skin organ culture model it was further shown that IL-1α can induce follicular hyperkeratinisation [12]. *Propionibacterium acnes* and oxidized lipids, both implicated in the pathogenesis of acne vulgaris, are also capable of directly inducing proinflammatory cytokine production in human epidermal keratinocytes and HaCaT cells [11, 27, 31]. Recently, Kistowska et al. [18] further demonstrated that IL-1β and the active processed forms of this cytokine are abundant in inflammatory acne lesions. Interestingly, *P. acnes* was found to activate the inflammasome in monocytes/macrophages. A role of IL-1 in mediating inflammation of the pilosebaceous unit is finally highlighted by the successful treatment of patients with PAPA syndrome with IL-1 antagonists [4]. Of note, IL-1 is primarily secreted by monocytes/macrophages [7] and the reported impact of this cytokine on keratinocyte proliferation is not specific for acroinfundibular keratinocytes. There is currently no evidence for an increased sensitivity of acroinfundibular keratinocytes to IL-1 or increased expression of this cytokine compared to interfollicular keratinocytes [17]. Thus, induction of IL-1 in acne may be regarded as a rather non-specific response of the pilosebaceous unit which is superimposed on increased sebaceous lipogenesis linked to increased insulin-like growth factor and androgen signaling of puberty [23].

l-Ascorbyl-2-phosphate (APS) is a stable antioxidant derived from vitamin C. In previous open-label studies 5 % sodium APS lotion appeared to have anti-inflammatory effects in patients with acne vulgaris. These beneficial effects of topical APS in acne were recently confirmed in a randomized double-blind controlled trial [32]. The precise mechanism by which topical APS elicits its anti-inflammatory effects in patients with acne vulgaris remains unknown. However, APS may exert suppressive effects on expression of distinct proinflammatory cytokines, which are known to be induced in a redox-sensitive manner, i.e., by activation of canonical nuclear factor-κB (NF-κB) signaling [10]. Notably, magnesium APS has previously been shown to exert protective and anti-inflammatory effects on UVB-treated mouse skin [19, 26], Moreover, various APS salts (e.g., sodium, calcium, magnesium) at doses ranging from 0.01 to 3 % are used in cosmetics in which they function as antioxidants and possibly also as weak anti-inflammatory cosmeceuticals [8]. However, despite of the mechanistic link between oxidative stress and inflammation in general the commonly used antiacne agent benzoyl peroxide is a strong oxidant.

Given the potential role of IL-1 in the pathogenesis of acne we investigated the molecular mechanism of proinflammatory cytokine induction and signaling in human sebocytes. To this end we used the immortalized human sebaceous gland cell line SZ95 derived from facial human sebaceous glands [35]. In addition, we tested the hypothesis as to whether APS can alter the impact of IL-1 and/or TNF-α (used as a control) on canonical NF-κB signaling and proinflammatory cytokine induction in these cells.

## Materials and methods

### Cell culture and reagents

SZ95 sebocytes were routinely maintained in Sebomed^®^ basal medium supplemented with 5 ng/ml human epidermal growth factor, 10 % fetal calf serum (FCS) (both from Biochrom, Berlin, Germany), 1 % l-glutamin, 1 % penicillin/streptomycin and 1 mM CaCl_2_ in a humidified atmosphere containing 5 % CO_2_ at 37 °C [35]. APS was purchased from Fluka (Taufkirchen, Germany), IL-1β from Biomol (Hamburg, Germany) and TNF-α from Immunotools (Friesoythe, Germany). Prior to stimulation with cytokines SZ95 sebocytes were deprived for 24 h in 0.1 % FCS (for all studies involving IL-1β) or 0 % FCS (for all studies with TNF-α).

### Cell viability assays

SZ95 sebocytes were seeded into 96-well tissue culture plates at a density of 10,000 cells/well. The following day cells were treated with APS at indicated doses in presence of 0.1 % FCS. Cell viability was measured by the XTT kit after 48 h (Roche, Mannheim, Germany). Quintuplicates were used for all treatments. Experiments were performed independently three times.

### Real-time RT-PCR

Cells were seeded into 3.5 cm Ø tissue culture dishes at a density of 2 × 10^5^ cells per dish. Following deprivation from FCS and stimulation as indicated established protocols were used for total RNA preparation, cDNA synthesis and real-time RT-PCR [22]. In short, total RNA was prepared by the RNeasy^®^ kit (Qiagen, Hilden, Germany) followed by DNAse I treatment cDNA synthesis using oligo-dT primers and RevertAid^®^ M-MuLV reverse transcriptase (Fermentas Life Sciences, Hanover, MD, USA). Real-time RT-PCR was performed with Taq polymerase (Promega, Mannheim, Germany) in a total volume of 20 μl with SYBR Green PCR Master Mix (Applied Biosystems, Foster City, CA) and a 200 nM concentration of intron-spanning primers. The primer sequences for IL-6 and IL-8 were identical as described before [22]. Primers for cyclooxygenase 2 (COX2) were sense 5′-TTGCTGGAACATGGAATTACCC-3′ and anti-sense 5′-GCATAAAGCGTTTGCG-GTACTC-3′. Reactions were carried out in duplicates in an ABI-prism 7000 sequence detector supplied with SDS 2.2 software (Applied Biosystems) using the following conditions: an initial activation step (2 min at 50 °C), a single denaturation step (15 min at 95 °C), followed by 40 cycles of 15 s at 95 °C and 60 s at 60 °C, and a final cycle of 15 s at 95 °C, 30 s at 60 °C and 15 s at 95 °C. Levels of gene expression in each sample were quantified applying the 2^−ΔΔCT^ method with glyceraldehyde-3-phosphate dehydrogenase (GAPDH) as an endogenous control. GAPDH primers were identical with those reported before [20].

### IL-6 and IL-8 protein analysis

Commercially available ELISAs from Ray Bio (Norcross, GA) were used to determine the amounts of IL-6 and IL-8 proteins generated by SZ95 sebocytes. Secreted levels of IL-6 and IL-8 were measured in culture supernatants of cells seeded into 3.5 cm Ø tissue culture dishes at a density of 200,000 cells per dish. All measurements were performed in duplicates according the manufacturer’s protocol. Experiments were performed independently at least three times.

### Detection of prostaglandin E_2_

A commercially available ELISA from Cayman (Ann Arbor, MI) was used to measure prostaglandin E_2_ (PGE_2_) in cell culture supernatants of SZ95 sebocytes. Cells were stimulated as outlined above followed by duplicate measurements according to the manufacturer’s protocol.

### Western immunoblotting

Serum-deprived SZ95 sebocytes were stimulated with IL-1β or TNF-α alone or in combination with APS as indicated. Cells were then either directly harvested in boiling Laemmli buffer or processed for subcellular fractionation as described before [22]. Protein concentrations in cytoplasmic and nuclear extracts were determined by the DC Protein assay kit (BioRad, Hercules, CA). Identical amounts of protein were resolved by 10 % SDS-PAGE, electrotransferred to PVDF membranes, blocked with 2 % blocking reagent (Roche, Mannheim Germany) and incubated with primary antibodies in 1 % blocking reagent overnight at 4 °C. Primary antibodies were anti-IκBα (IMGENEX, San Diego, CA), NF-κB/p65 (Biomol), anti-phospho-IκBα, anti-COX2, anti-phospho-p38 mitogen-activated protein kinase (MAPK) and anti-phospho-p42/p44 MAPK1/2 (all from Cell Signaling, Frankfurt am Main, Germany). Equal loading of protein lysates for the detection of phosphorylated p38 MAPK and phospho-p42/p44 MAPK1/2 was confirmed by stripping and reprobing the membranes with anti-p38 MAPK and anti-p42/p44 MAPK1/2 antibodies (both from Cell Signaling). In some experiments, phorbol 12-myristate 13-acetate (PMA, 50 ng/ml) was used as positive control. After washing, membranes were incubated with secondary horseradish peroxidase conjugated antibodies (Amersham-Pharmacia) in 1 % blocking reagent for 1 h at room temperature. Bound antibodies were detected using the ECL Plus chemiluminescent reagent (Amersharm-Pharmacia). Identical nuclear protein loading was confirmed by stripping the blot and reprobing it with anti-α-tubulin antibodies (Calbiochem, Schwalbach, Germany) or with an antibody against the TATA-binding protein (TBP; Abcam, Milton, UK).

### Immunofluorescence analysis

SZ95 sebocytes were seeded into 8-well chamber slides at a density of 10,000 cells per well. After deprivation from FCS as outlined above and stimulation with IL-1β or TNF-α alone and in combination with APS stand cells were fixed with methanol for 30 min at −20 ^o^C. Non-specific binding was then blocked with 5 % donkey serum for 1 h at room temperature followed by incubation for 1 h with a rabbit polyclonal antibody against NF-κB p65 (Santa Cruz Biotechnology Inc., San Diego, CA). Bound antibodies were detected with a donkey anti-rabbit antibody coupled to Texas Red (1:1500; Dianova, Hamburg, Germany). Cells were imaged and examined with a fluorescence microscope (Carl Zeiss, Oberkochen, Germany). Red fluorescence was excited using the TX3 filter (530–585 nm). Slides were finally imaged by fluorescence microscopy (Carl Zeiss, Jena, Germany).

### Densitometry

Western blot images were processed for densitometric analysis using the freely available software Image J (http://rsbweb.nih.gov/ij). Mean intensities were corrected for background followed by normalization with the corresponding housekeeping gene products α-tubulin, nuclear TBP, or non-phosphorylated proteins (p38, ERK1/2) in case of phosphorylated kinases. For multiple experiments and statistical analysis mean intensities of control samples (non-treated, cytokine- or PMA-treated cells) were set as 1 and changes of intensity were calculated as fold increase or decrease from control.

### Data analysis

All experiments were performed at least three times. Data were calculated as mean ± SD followed by statistical analysis using the Student’s *t* test for unpaired experiments. A *p* value of <0.05 was considered statistically significant.

## Results

### Effects of APS on IL-6 and IL-8 mRNA expression in SZ95 sebocytes

**Fig. 1:**
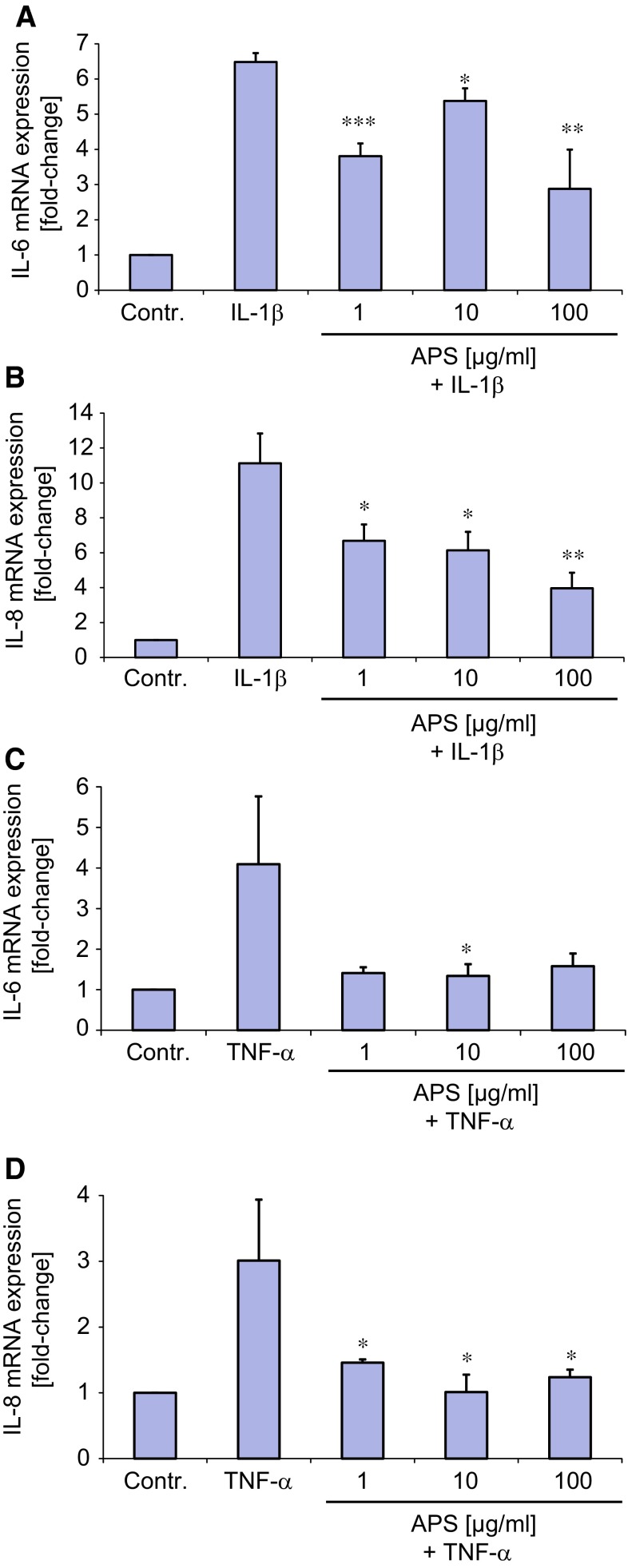
APS suppressed IL-1β- and TNF-α-mediated mRNA expression of IL-6 (**a**, **c**) and IL-8 (**b**, **d**) in SZ95 sebocytes. Cells were treated with IL-1β (0.1 ng/ml), TNF-α (10 ng/ml) alone or in combination with APS at indicated doses for 6–8 h followed by real-time RT-PCR analysis. ***p < 0.001, **p < 0.01, *p < 0.05 vs. IL-1β or TNF-α; *n* = 3

We first determined the effect of APS at various doses on the cell viability of sebocytes in vitro using XTT test. Incubation of SZ95 sebocytes with APS for 48 h revealed that doses of 1–1000 µg/ml were non-cytotoxic as shown in online resource 1 (Supplemental Figure 1). Next, the effect of APS on IL-6 and IL-8 mRNA expression was investigated by real-time RT-PCR after stimulation of SZ95 sebocytes with IL-1β or TNF-α. Previously, we had established that IL-1β has a time- and dose-dependent effect on the steady-state levels of both IL-6 and IL-8 mRNAs in SZ95 sebocytes [22]. Therefore, we used IL-1β at 0.1 ng/ml, which reproducibly and robustly increased the mRNA amounts of both IL-6 and IL-8 mRNA in SZ95 sebocytes, i.e., ~six- to tenfold over control (*p* < 0.001) (Fig. 1a, b). With regard to TNF-α only very high doses of this cytokine were capable of reproducibly increasing the mRNA levels of both IL-6 and IL-8 (online resource 1, Supplemental Figure 2a, b). Thus, for all subsequent studies TNF-α at 10 ng/ml was used. Interestingly, APS at all tested doses (1–100 µg/ml) reduced IL-1β-mediated expression of both IL-6 and IL-8 mRNA. This effect of APS appeared to be dose-dependent for IL-8 mRNA expression with an IC50 of ~100 µg/ml (Fig. 1a, b). Likewise, APS suppressed TNF-α-mediated mRNA expression of both IL-6 and IL-8 at the majority of tested doses albeit no clear-cut dose-kinetic effect could be detected (Fig. 1c, d). APS tested at various doses per se did not change the mRNA expression levels of IL-6 and IL-8 (data not shown).

### Impact of IL-1β, TNF-α and APS on NF-κB signaling in SZ95 sebocytes

**Fig. 2:**
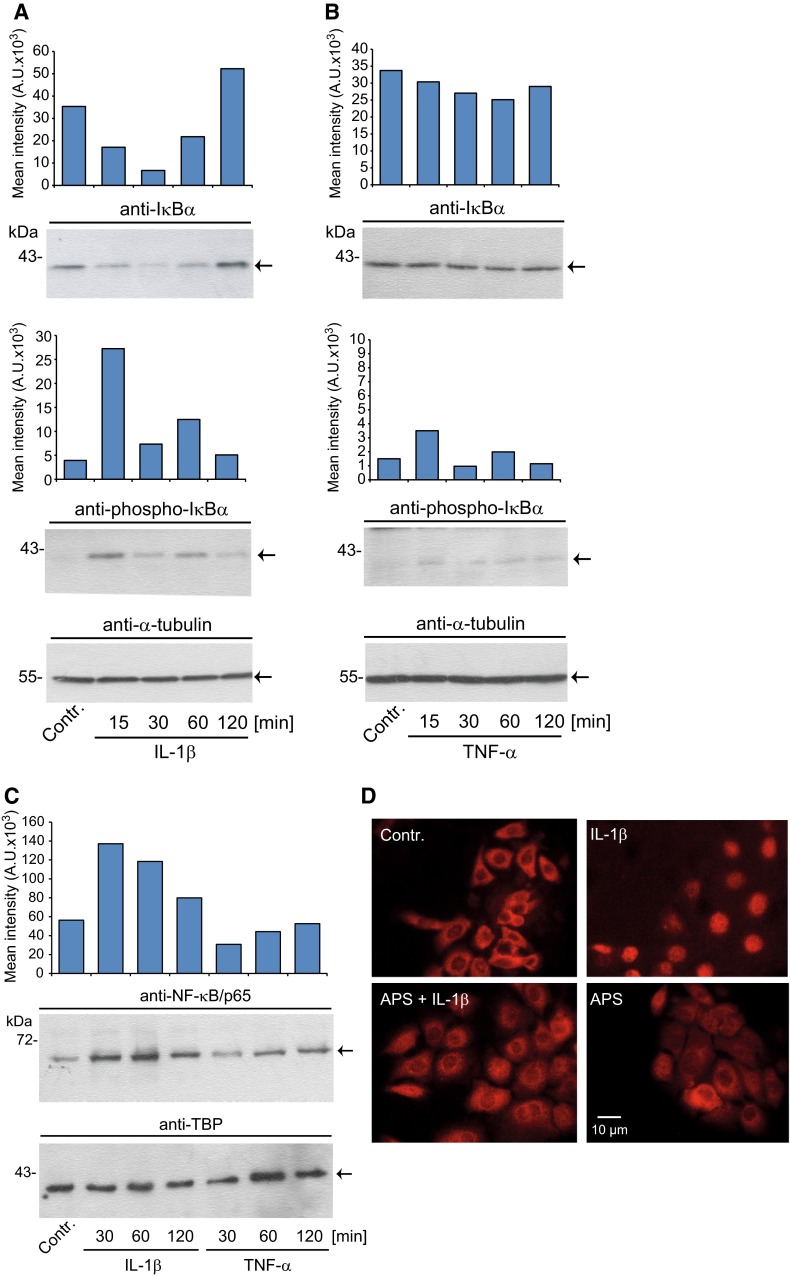

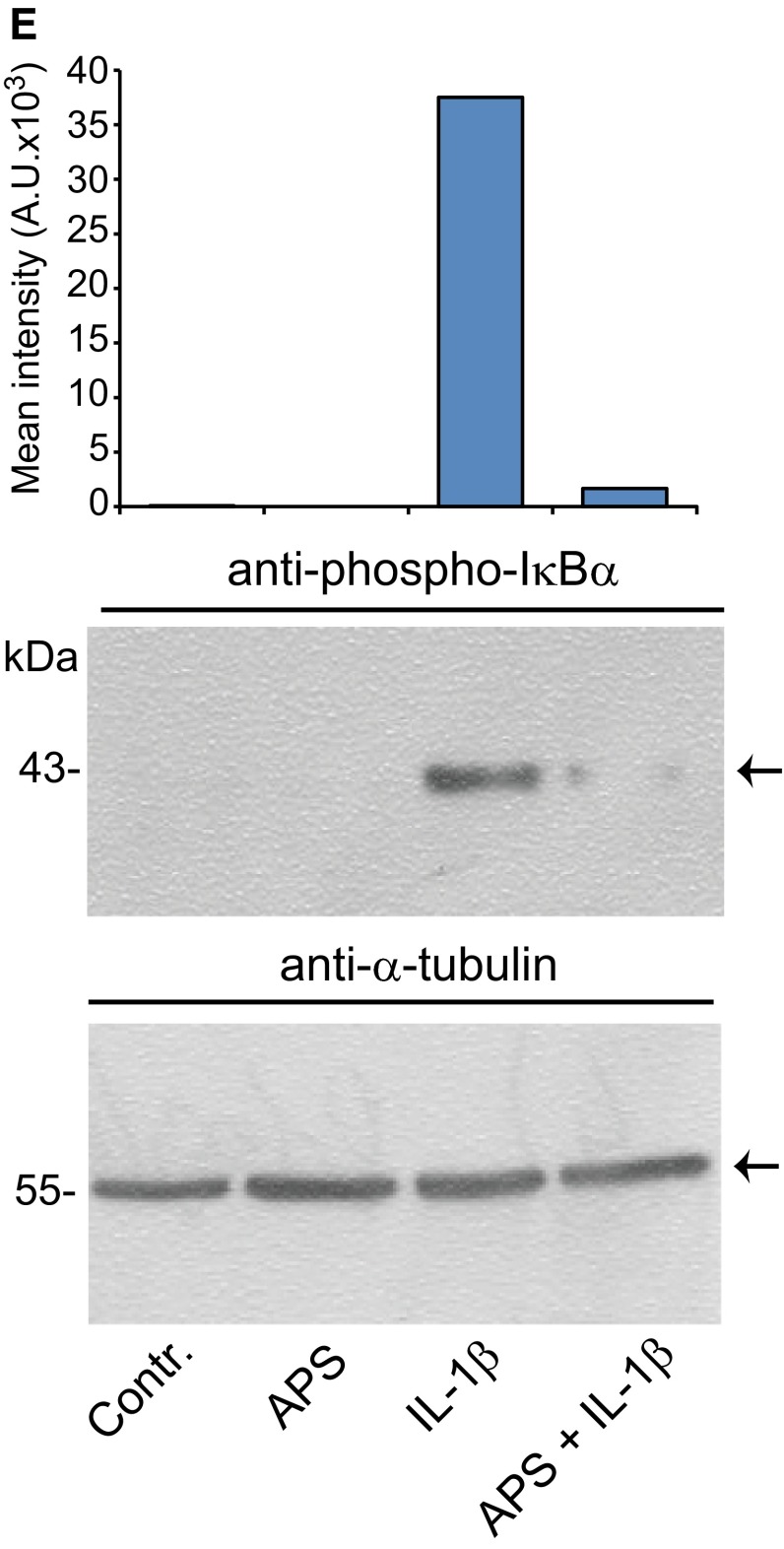
Impact of IL-1β, TNF-α and APS on NF-κB activation in SZ95 sebocytes. Time kinetics of IL-1β- (**a**) and TNF-α- (**b**) mediated IκBα phosphorylation and protein degradation. Cells were treated with IL-1β (0.1 ng/ml) or TNF-α (10 ng/ml) as indicated followed by Western immunoblotting of total cell lysates with antibodies against phosphorylated and total IκBα. To ensure equal protein loading membranes were reprobed with an anti-tubulin-α antibody. Expression of phosphorylated and total IκBα was quantified by densitometry. Time kinetic analysis of nuclear accumulation of NF-κB/p65 after stimulation with IL-1β or TNF-α (**c**). Nuclear extracts were processed by Western immunoblotting using antibodies against NF-κB/p65. Nuclear protein loading was controlled by reprobing with an anti-TBP antibody. Expression of phosphorylated and total IκBα was quantified by densitometry. APS reduces nuclear translocation of NF-κB/p65 following stimulation with IL-1β (**d**). Nuclear translocation of NF-κB/p65 was assessed by immunofluorescence 30 min after stimulation. *Panels* depict representative images of at least three independent experiments with identical results. APS attenuates NF-κB activation as shown by reduced phosphorylation of IκBα (**e**). Phosphorylation of IκBα was determined by Western immunoblotting as outlined above. Expression of NF-κB/p65 was quantified by densitometry

Since IL-6 and IL-8 expression is well known to be regulated by canonical NF-κB signaling we next wondered whether APS affects the activation of this key regulator of inflammatory cytokine responses. Upon stimulation with IL-1β or TNF-α NF-κB is activated by phosphorylation of IκBα followed by proteasomal degradation of this protein. Homo- or heterodimers of NF-κB/p65 or NF-κB/p50 translocate then to the nucleus and bind to NF-κB consensus elements, for example., in the promoters of IL-6 and IL-8. However, comparative studies on the activation of NF-κB by IL-1β and TNF-α have not been performed to the best of your knowledge in SZ95 sebocytes. Stimulation of these cells with IL-1β led to a time-dependent phosphorylation of IκBα as shown by Western immunoblotting (Fig. 2a, lower panel). This IL-1β-mediated phosphorylation was statistically significant 15 min after stimulation (mean increase: 5.94 ± 1.51-fold, *n* = 3, *p* < 0.01). In contrast, no significant phosphorylation of IκBα was detected upon stimulation of SZ95 sebocytes with TNF-α (Fig. 2b, lower panel). In accordance with these findings the protein amounts of IκBα were time dependently reduced (statistically significant at 30 min; mean decrease: 0.18 ± 0.03-fold, *p* < 0.0001, *n* = 3) after IL-1β but not after TNF-α treatment **(**Fig. 2a, b, upper panel). Time kinetic analyses using nuclear extracts further revealed that the amounts of NF-κB/p65 accumulated in the nucleus over time in response to IL-1β (statistically significant at 30 min; mean increase: 2.36 ± 0.33-fold, *n* = 3, *p* < 0.01) but not after TNF-α (Fig. 1c). Next, the impact of APS was assessed in SZ95 sebocytes using two NF-κB activation read-outs after stimulation with IL-1β. Nuclear translocation of NF-κB/p65 was reduced by APS as shown by immunofluorescence analysis (Fig. 2d). As shown by Western immunoblotting APS (10 µg/ml) also markedly reduced the extent of IκBα phosphorylation in cells stimulated with IL-1β (mean decrease: 0.37 ± 0.02-fold, *p* < 0.05, *n* = 4) (Fig. 2e). As expected, APS did neither alter the expression or phosphorylation of IκBα nor the immunolocalization of NF-κB/p65 in SZ95 sebocytes treated with TNF-α (data not shown).

### APS does not alter IL-1β and TNF-α-mediated secretion of both IL-6 and IL-8 by SZ95 sebocytes

**Fig. 3:**
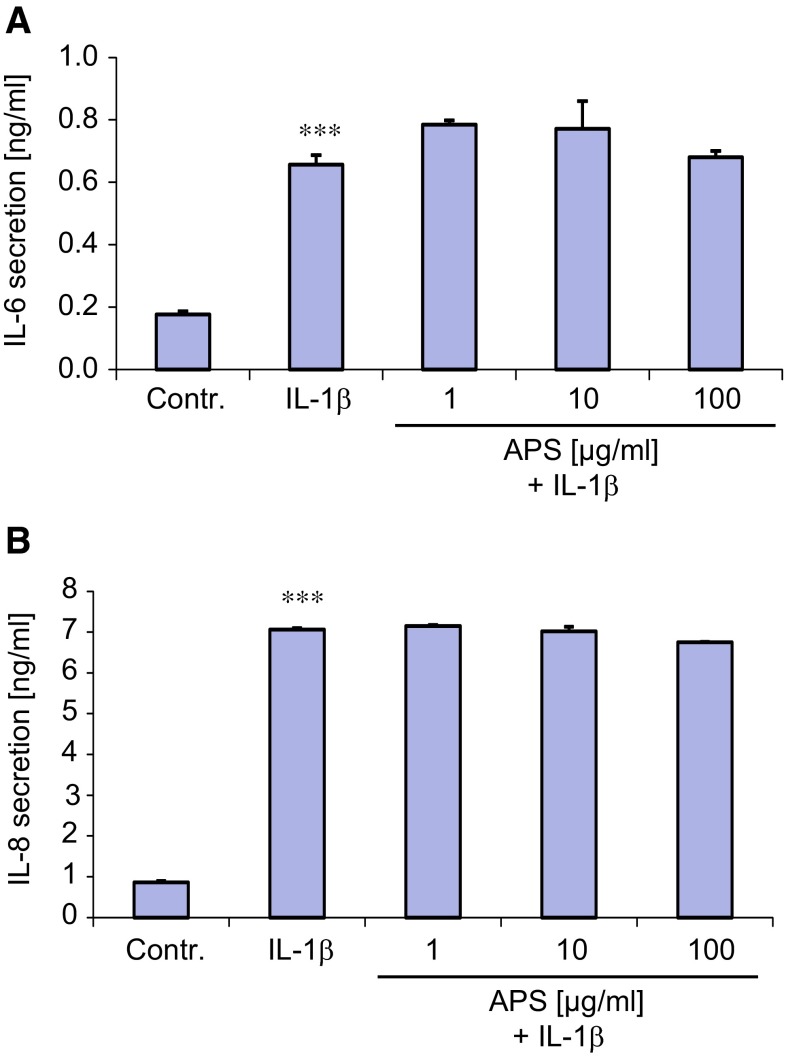
Effect of APS on IL-1β- and TNF-α-mediated IL-6 (**a**) and IL-8 (**b**) secretion by SZ95 sebocytes. Cells were stimulated with IL-1β (0.1 ng/ml), TNF-α (10 ng/ml) alone or in combination with APS at indicated doses for 24 h followed by ELISA, *n* = 3

The findings show that APS reduces both IL-1β- and TNF-α-mediated mRNA expression of IL-6 and IL-8 and is also capable of attenuating IL-1β-mediated NF-κB activation suggested that this antioxidant may also suppress the secretion of IL-6 and IL-8 by SZ95 sebocytes. Thus, the levels of IL-6 and IL-8 were determined by ELISA after stimulation of SZ95 sebocytes with IL-1β and TNF-α alone and in presence of different concentrations of APS. Stimulation with IL-1β (0.1 ng/ml) for 24 h resulted in a significant increase in the secreted amounts of both IL-6 and IL-8 (*p* < 0.001) (Fig. 3a, b). However, in contrast to the corresponding mRNA levels (Fig. 1a, b) APS at doses ranging from 1 to 100 µg/ml failed to alter IL-1β-mediated secretion of both cytokines in SZ95 sebocytes compared with IL-1β alone (Fig. 3a, b). Stimulation of cells for shorter time periods, i.e., for 3 and 12 h, likewise did not disclose a modulatory effect of APS on IL-1β-mediated secretion of both cytokines. As expected, APS per se did not affect secretion of IL-6 and IL-8 as determined by stimulation of cells with one representative APS dose (10 µg/ml) (data not shown). When supernatants of SZ95 sebocytes exposed to TNF-α alone or in combination with APS were further tested we could not detect any significant changes in the amount of both IL-6 and IL-8 compared to non-treated controls (data not shown).

### APS does not alter IL-1β-mediated activation of p38 MAPK in SZ95 sebocytes

**Fig. 4:**
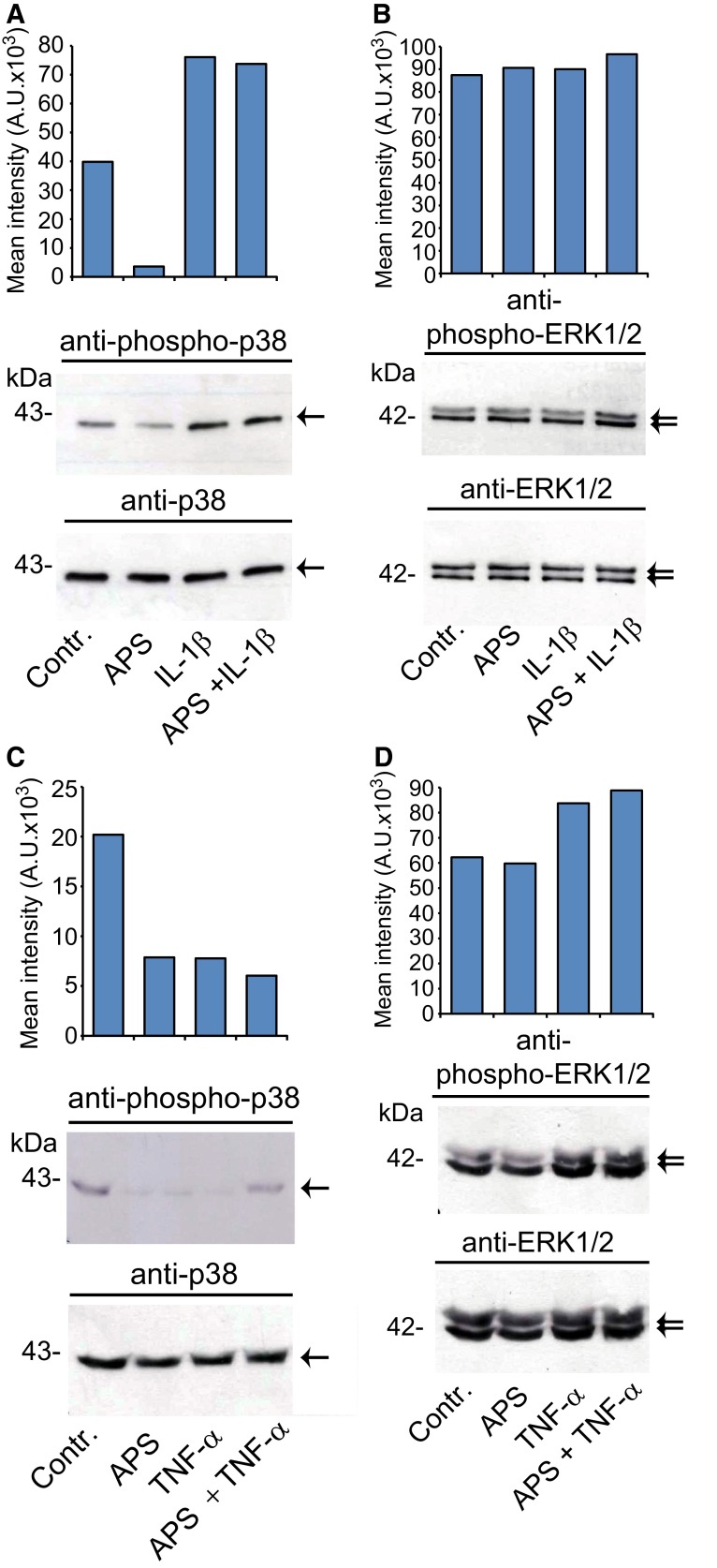
Effects of IL-1β, TNF-α and APS on p38 MAPK (**a**, **c**) and p42/p44 ERK1/2 (**b**, **d**) phosphorylation in SZ95 sebocytes. Cells were stimulated with IL-1β (0.1 ng/ml) and TNF-α (10 ng/ml) alone or in combination with APS (1 mg/ml) for 30 min followed by Western immunoblotting with phosphospecific antibodies against p38 MAPK and p42/p44 ERK1/2. To ensure equal protein loading membranes were reprobed with antibodies against total p38 MAPK and p42/p44 ERK1/2. Expression of phosphorylated kinases was quantified by densitometry. *Panels* depict representative images of 3 independent experiments with similar results

Puzzled by the observation that APS attenuates IL-1β-mediated NF-κB activation (Fig. 2c–e) and NF-κB-dependent gene expression but does neither suppresses IL-6 nor IL-8 secretion we wondered as to whether this antioxidant may alter the activation of MAPKs in SZ95 sebocytes. Detailed molecular studies from others revealed that distinct members of the MAPK family, especially p38 MAPK, are crucially involved in the regulation of IL-8 secretion in the context of NF-κB-mediated IL-8 gene expression [14]. Thus, we checked the activated state of p38 MAPK in total cell lysates of SZ95 sebocytes by Western immunoblotting using phosphospecific antibodies. Treatment with IL-1β induced significant phosphorylation of p38 MAPK in SZ95 sebocytes (mean increase: 1.64 ± 0.31-fold, *p* < 0.05, *n* = 3). However, concomitant incubation with APS did not alter the extent of p38 MAPK phosphorylation (Fig. 4a). Interestingly, APS per se decreased p38 MAPK phosphorylation (mean decrease: 0.30 ± 0.28-fold, *p* < 0.05, *n* = 3). The lack of an effect of APS on IL-1β-mediated p38 MAPK phosphorylation was further observed when cells were treated with the artificial protein kinase C activator PMA (online resource 1, Supplemental Figure 3a). Here, PMA increased p38 MAPK phosphorylation without further modulation by APS. In contrast to these findings we could not detect any changes in the phosphorylated state of p42/p44 MAPK1/2/ERK1/2 (extracellular signal-regulated kinase 1/2) in SZ95 sebocytes by IL-1β compared to non-stimulated cells (Fig. 4b). Likewise PMA did not alter constitutive p42/p44 ERK1/2 phosphorylation in SZ95 sebocytes (online resource 1, Supplemental Figure 3b). Given the regulatory role of p38 MAPK on IL-8 secretion we also wondered whether phosphorylation of p38 MAPK would be modulated by TNF-α or APS in SZ95 sebocytes. Treatment with either substances (or both) interestingly reduced the phosphorylated state of this kinase (for TNF-α mean decrease: 0.47 ± 0.18-fold, *p* < 0.05, *n* = 4) (Fig. 4c). In contrast, neither TNF-α nor TNF-α plus APS had significant effects on ERK1/2 phosphorylation in SZ95 sebocytes in multiple experiments (Fig. 4d).

### Impact of IL-1β, TNF-α and APS on COX2 expression and PGE_2_ secretion in SZ95 sebocytes

**Fig. 5:**
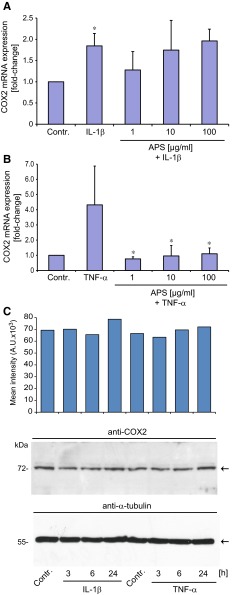
Impact of IL-1β, TNF-α and APS on COX2 mRNA (**a**, **b**) and protein expression (**c**) in SZ95 sebocytes. Expression of COX2 transcripts was determined by real-time RT-PCR following stimulation of cells with either IL-1β or TNF-α (10 ng/ml) alone or in combination with APS for 8 h. *p < 0.05 vs. control for IL-1β, *n* = 3; *p < 0.05 vs. TNF-α, *n* = 4. Expression of COX2 protein was assessed by Western immunoblotting following stimulation of cells with IL-1β or TNF-α as indicated. Expression of COX2 protein was quantified by densitometry. *Panels* are representative images of at least three independent experiments with identical results

We finally investigated the effects of IL-1β, TNF-α and APS on the expression of COX2 and PGE_2_ in SZ95 sebocytes. PGE_2_ has been implicated in the pathogenesis of acne and has also been suggested to be a viable target for the treatment of this disease [28, 33, 34]. Previous studies had revealed the presence of the full enzymatic machinery for the biosynthesis of leukotriene B4 and PGE_2_ in sebaceous glands of acne patients and SZ95 sebocytes, respectively [2]. Albeit it was shown that arachidonic acid stimulated the biosynthesis of PGE_2_ in SZ95 sebocytes, the impact of IL-1β and TNF-α on COX_2_ expression and PGE_2_ secretion is unknown. As demonstrated by real-time RT-PCR both IL-1β and TNF-α had upregulating effects on the mRNA level of COX2 (*p* < 0.05, *n* = 3 for IL-1β; *p* < 0.05, *n* = 4 for TNF-α) (Fig. 5a, b). Interestingly, APS at all tested doses (1–100 µg/ml) suppressed the effect of TNF-α but not of IL-1β on COX2 mRNA expression **(**Fig. 5a, b). However, the modulating effects of TNF-α, IL-1β and also of APS—in case of TNF-α-mediated COX2 mRNA expression—were not associated with altered COX2 protein levels in SZ95 sebocytes (Fig. 5c). This lack of an effect of IL-1β and TNF-α on COX2 was confirmed by measuring the amounts of PGE_2_ in the supernatants of SZ95 sebocyte cultures. Basal secretion of PGE_2_ within 24 h was 73.19 ± 3.6 pg/ml per 100,000 cells. Neither IL-1β, TNF-α nor APS had any impact on the levels of PGE_2_. Further, different time points, i.e., stimulation with the above agents for 1, 3 and 16 h, did not have any effect on the secretion of this short-term mediator (data not shown).

## Discussion

In this study we have examined the impact of two key proinflammatory cytokines, IL-1β, TNF-α, but also of the antioxidant APS on IL-6 and IL-8 expression and NF-κB signaling in the human sebaceous gland cell line SZ95. While IL-1 has been shown to be involved in inflammatory processes in human sebocytes in vivo and in vitro, TNF-α is probably not a mediator of inflammation in these cells. Our hypothesis to test APS in this context was first based on the clinical observation that APS has beneficial effects in patients with acne vulgaris [32]. Second, APS is an antioxidative vitamin C derivative. Previous studies revealed that vitamin C is capable of reducing NF-κB-dependent induction of IL-8 gene expression by TNF-α in the human histiocytoma lymphoma cell line U937 [30]. Moreover, vitamin C inhibited activation of NF-κB and NF-κB-dependent gene IL-8 expression by TNF-α in the endothelial cell line ECV304 and in primary human umbilical vascular endothelial cells [3]. Bowie and O´Neill [3] disclosed that vitamin C blocked IL-1α and TNF-α-mediated degradation and phosphorylation of IκBα due to inhibition of IκB kinase activation. However, in both of these studies it was not described if vitamin C also inhibited IL-8 protein expression or secretion. Our findings on the inhibitory effect of APS on IL-1β in SZ95 sebocytes are in accordance with the previously described effect of vitamin C in U937 and endothelial cells. Accordingly, attenuation of NF-κB signaling in SZ95 sebocytes was demonstrated by reduced phosphorylation of IκBα and reduced nuclear accumulation of NF-κB/p65 following stimulation with IL-1β. Interestingly, TNF-α, which also induces IL-8 gene expression, did not appear to affect NF-κB activation in SZ95 sebocytes as evidenced by the lack of IκBα degradation, phosphorylation and no detectable nuclear accumulation of p65. Thus, NF-κB activation in SZ95 sebocytes seems to be a specific IL-1-mediated event, indicating a major role of IL-1 in sebocyte inflammatory processes, as previously shown. Molecular studies revealed that IL-8 gene expression is extremely complex and coordinated by at least three events: (a) derepression of the gene promoter; (b) transcriptional activation of the gene by NF-κB and JUN N-terminal protein kinase pathways; and (c) stabilization of the mRNA by the p38 MAPK pathway [13]. For IL-6 gene expression activation of the transcription nuclear factor-IL-6 (NF-IL6) is also critical [1]. IL-8 protein expression and secretion was found to depend on distinct MAPK kinase kinases which can activate NF-κB, i.e., stress-activated protein kinase/jun N-terminal kinase and p38 MAP kinases [14]. Indeed, our study is limited as we have not examined the activation of stress-activated protein kinase/jun N-terminal kinase by IL-1, TNF-α or APS. With regard to p38 activation others could detect phosphorylation of this kinase after TNF-α treatment of SZ95 sebocytes within 5–10 min followed by a rapid decline to basal levels [6]. This is in contrast to our findings as we noticed even reduced p38 phosphorylation after TNF-α and/or APS after 30 min of treatment. While the reduction of p38 phosphorylation by APS may be related to the antioxidative effects of this vitamin C derivative the reason for the reduction of p38 after TNF-α is unclear but may be due to compensatory dephosphorylation after early transient phosphorylation that we may have missed by studying only the 30-min time point. Interestingly, other noticed even increased p38 phosphorylation after treatment of ECV304 cells with vitamin C [3] which may be related to higher doses of this molecule compared to our studies and cell type-specific differences. In accordance with Choi et al. [6], however, SZ95 sebocytes displayed constitutive phosphorylation of both p38 and EKR1/2 even after FCS starvation. Notably, the latter authors likewise did not find any modulation of ERK1/2 phosphorylation by TNF-α which is supported by our studies. Whether the lack of a IL-6 or IL-8 secretion after TNF-α treatment of SZ95 is related to the extent or duration of p38 phosphorylation needs to be addressed in future studies. Our observation, APS did not affect IL-1β-mediated phosphorylation of p38 MAP kinase, may explain why this agent—despite of its effect on NF-κB gene expression—failed to reduce IL-1β-mediated secretion of IL-6 and IL-8.

The finding shows that APS neither reduces IL-6 and IL-8 protein expression nor affects PGE_2_ secretion in SZ95 sebocytes suggests another mechanism of action of this agent in patients with acne vulgaris. Indeed, the SZ95 sebaceous gland cell line has certain limitations as being an in vitro model for acne. Moreover, the administered amounts of APS in patients with acne vulgaris are markedly higher (5 %) than the doses used in our in vitro studies. Interestingly, it was reported that the quantities of lipid peroxides caused by oxidation of sebum, NF-κB/p65 protein and IL-1α are significantly higher in comedones than those in stratum corneum [29]. Moreover, the antioxidant glutathione was recently found to be lower in the stratum corneum of patients with acne than in healthy subjects [17]. In light of these observations and the modulating effect of APS on NF-κB activation other target genes than IL-6 or IL-8 may be affected by APS at the protein level in vivo in acne patients. Thus, it will be very informative to measure lipid peroxides, glutathione, and NF-κB/p65 protein in the skin of acne patients treated with APS to further elucidate the mechanism of action of this antiacne compound. Moreover, it is possible that APS exerts its beneficial effects in acne patients via modulating activation of the mechanistic/mammalian target of rapamycin complex 1 (mTORC1) which is a master regulator of cellular metabolism. Recently, it was proposed by Melnik and Schmitz [24] that therapeutic effects of antiacne agents are mediated by activation of FoxO1 and inhibition of mTORC1. Since proinflammatory NF-κB activation inhibits tuberous sclerosis complex 1 and thereby activates mTORC1 a potential link between APS and mTORC signaling may exist.

## Electronic supplementary material

Supplementary material 1 (PDF 203 kb)
