# Supplementary material for: l-Ascorbyl-2-phosphate attenuates NF-κB signaling in SZ95 sebocytes without affecting IL-6 and IL-8 secretion
Source: Arch Dermatol Res. 2015 Apr 18;307(7):595–605. doi: 10.1007/s00403-015-1565-z (PMC4540786; doi:10.1007/s00403-015-1565-z)
Supplement: Supplementary file 1 — Supplementary material 1 (PDF 203 kb) [file 403_2015_1565_MOESM1_ESM.pdf]

Online resource 1

L-Ascorbyl-2-phosphate attenuates NF-κB signaling in SZ95 sebocytes without affecting IL-6 and IL-8 secretion

Archives of Dermatological Research

Hiroshi Ikeno[1], Mara Apel[2], Christos Zouboulis[3], Thomas A. Luger[2], Markus Böhm[2]

[1]Ikeno Clinic of Dermatology & Dermatologic Surgery, Tokyo, Japan

[2]Dept. of Dermatology, University of Münster, Münster, Germany

[3]Depts. of Dermatology, Venereology, Allergology and Immunology, Dessau Medical Center, Dessau, Germany

[*]Address correspondence and reprint requests to:

Hiroshi Ikeno, MD
Ikeno Clinic of Dermatology & Dermatologic Surgery
Ginza 1-14-4, 3F, Chuo-ku
Tokyo
Japan
Tel.: +81-3-3538-1344
Fax: +81-3-3538-1355
E-mail: ikenoderma@nifty.com

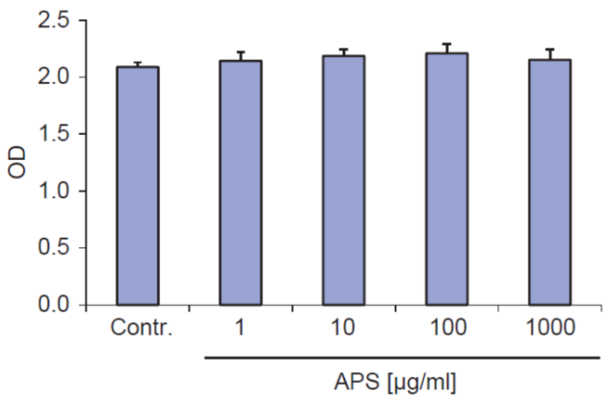

Supplmental Figure 1: Impact of APS on cell viability of SZ95 sebocytes as determined by XTT test. Cells were incubated for 48 hrs with APS at different concentraions as indicated. n=3.

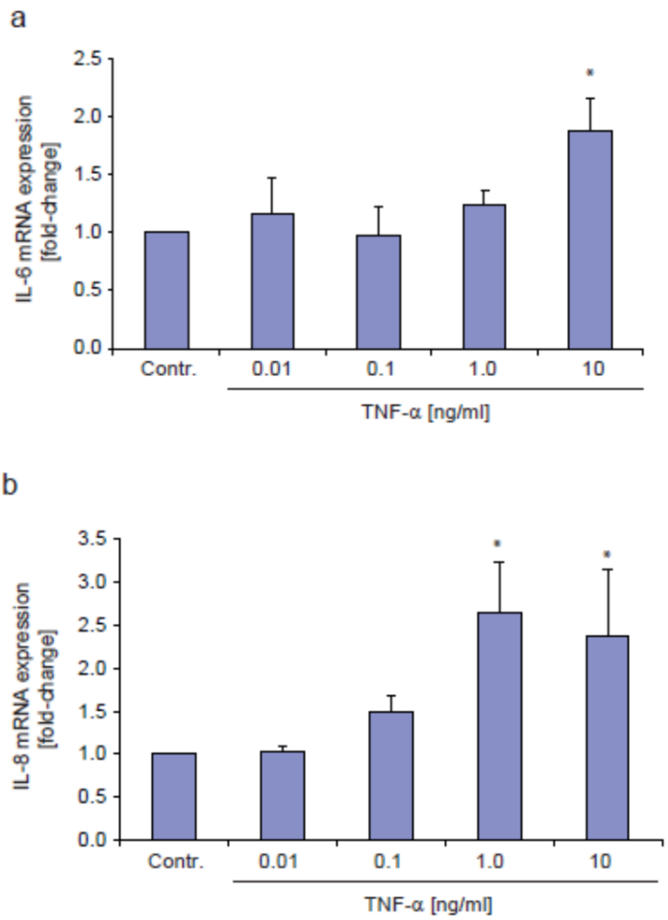

Supplmental Figure 2: Effect of TNF-α on IL-6 **(a)** and IL-8 mRNA expression **(b)** in SZ95 sebocytes as determined by real-time RT-PCR analysis. Cells were treated for 8 hrs as indicated. n=3, *$p<0.05$ vs. control.

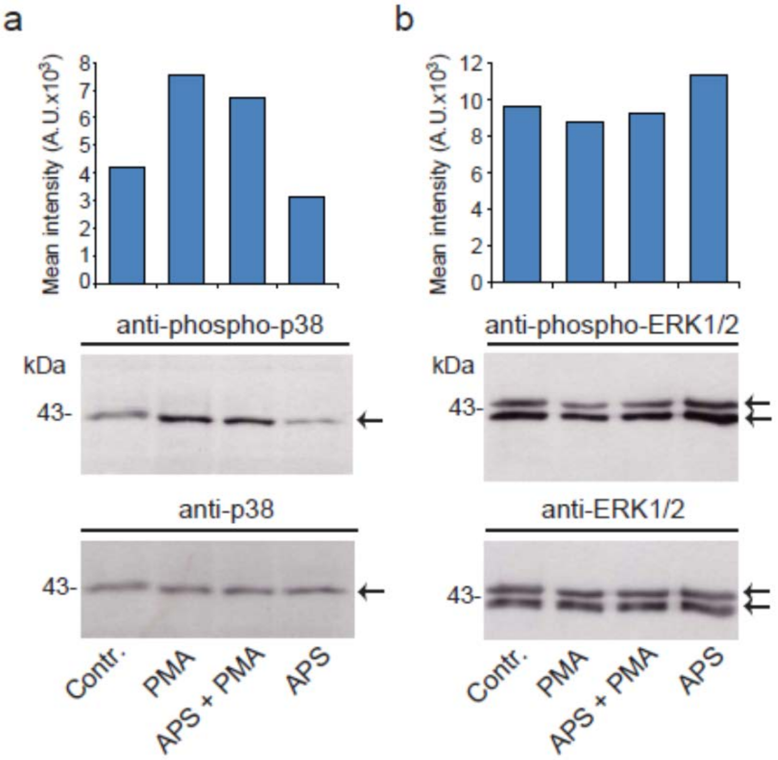

Supplmental Figure 3: Effect of PMA and APS on p38 MAPK **(a)** and ERK1/2 phosphorylation **(b)** in SZ95 sebocytes. Cells were stimulated with PMA (50 ng/ml) alone or in combination with APS (1 mg/ml) for 30 min followed by Western immunoblotting with phosphospecific antibodies against p38 MAPK and p42/p44 ERK1/2. To ensure equal protein loading membranes were reprobed with antibodies against total p38 MAPK and p42/p44 ERK1/2. Expression of phosphorylated kinases was quantified by densitometry. Panels depict representative images of 2 independent experiments with similar results.
